# Supplementary material for: Ultrahigh energy-dissipation elastomers by precisely tailoring the relaxation of confined polymer fluids
Source: Nat Commun. 2021 Jun 14;12:3610. doi: 10.1038/s41467-021-23984-2 (PMC8203694; doi:10.1038/s41467-021-23984-2)
Supplement: Supplementary file 1 — Supplementary Information [file 41467_2021_23984_MOESM1_ESM.pdf]

Supplementary Information

Ultrahigh energy-dissipation elastomers by precisely tailoring the relaxation of confined polymer fluids

Jin Huang[1], Yichao Xu[1,2], Shuanhu Qi[1,3], Jiajia Zhou[1,3]*, Wei Shi[1], Tianyi Zhao[1] and Mingjie Liu[1,2,3,4]*

[1] Key Laboratory of Bioinspired Smart Interfacial Science and Technology of Ministry of Education, School of Chemistry, Beihang University, Beijing 100191, P. R. China.

[2] Beijing Advanced Innovation Center for Biomedical Engineering, Beihang University, Beijing, 100191, P. R. China.

[3] International Research Institute for Multidisciplinary Science, Beihang University, Beijing, 100191, P. R. China.

[4] Research Institute of Frontier Science, Beihang University, Beijing, 100191, P. R. China.

* Corresponding author: E-mail: jjzhou@buaa.edu.cn, liumj@buaa.edu.cn

Supplementary Figures

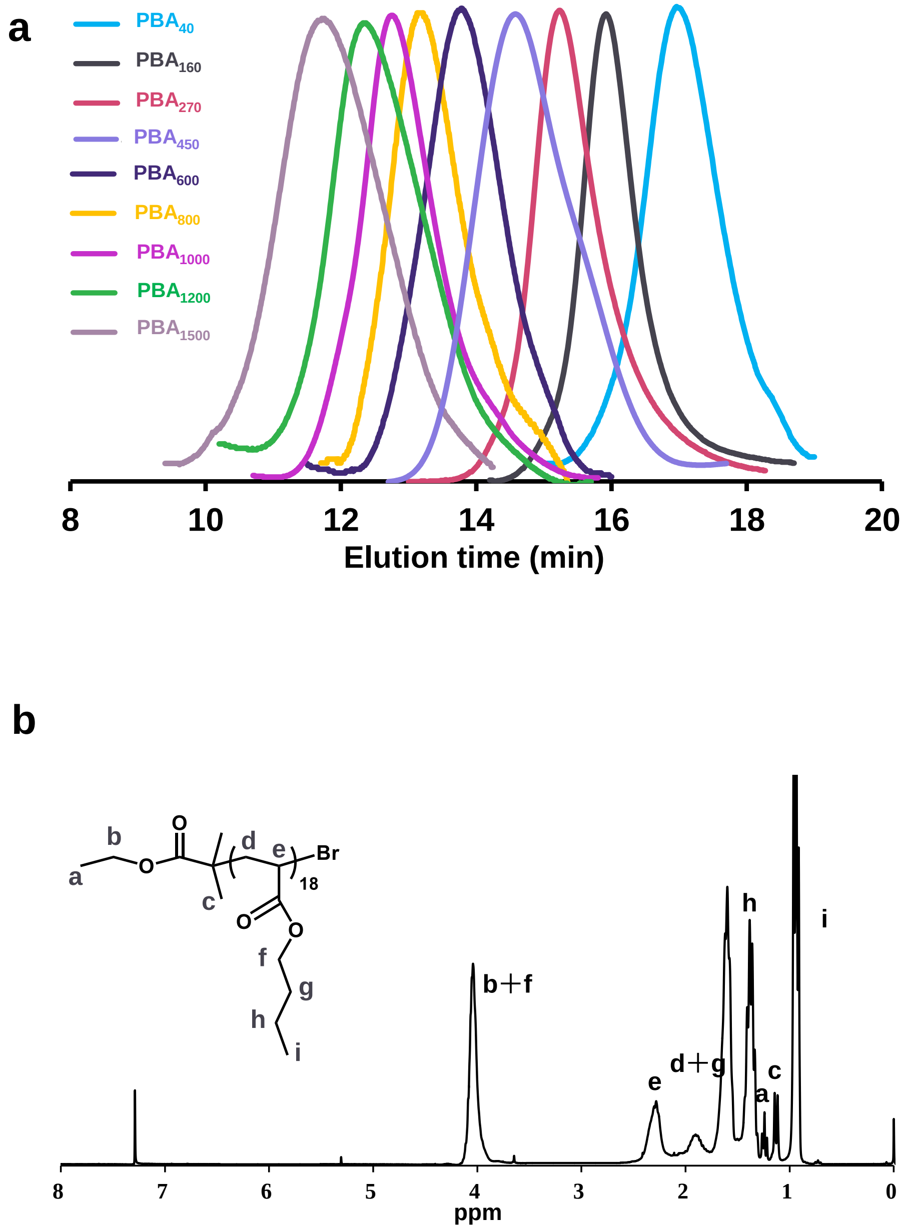

Supplementary Fig. 1 | Molecular structural characterization. (a) GPC traces of the synthesis of PBA fluids. **(b)** ^{1}H-NMR spectra of PBA_{40} melt (400MHz, $CDCl_3$): 4.05 ppm (-OCH_2-), 2.27 ppm (-CH_2-), 1.85-1.60 ppm (-CH_2-), 1.40-0.95 ppm (CH_3-CH_2-).

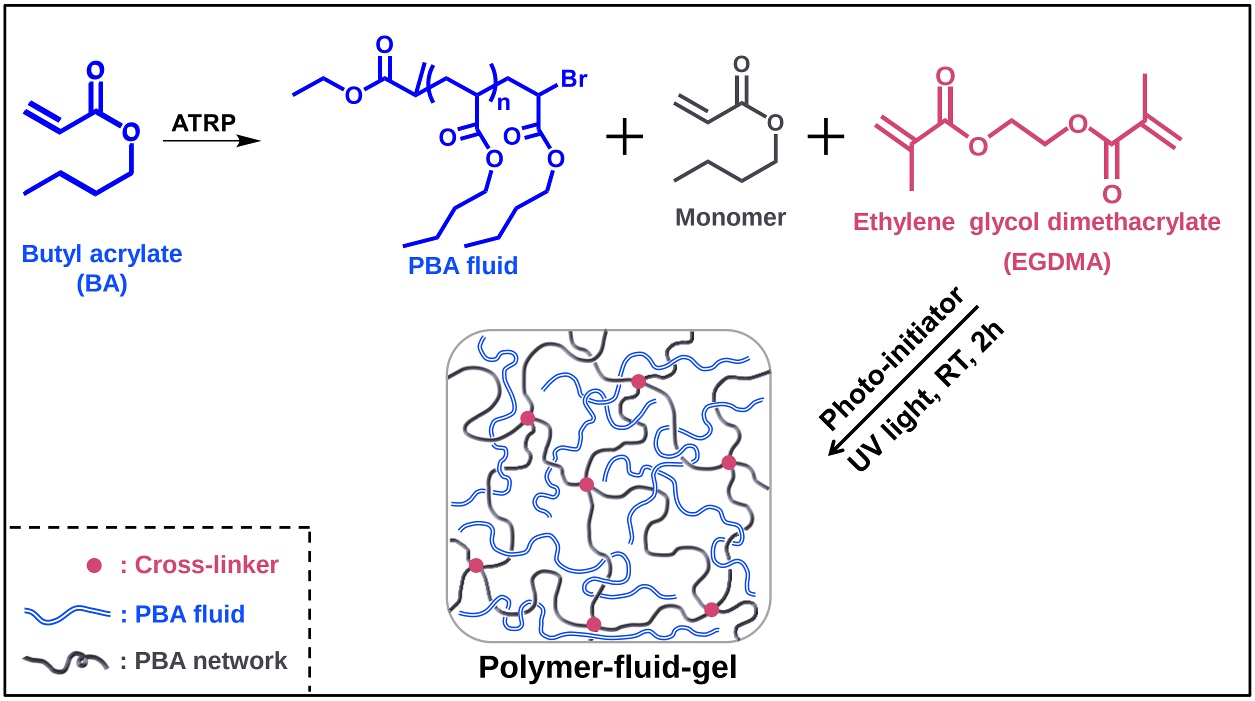

Supplementary Fig. 2 | Synthesis process of PFGs. All PFGs were prepared by photo-initiated radical polymerization. The initial reaction mixtures contained: monomer, PBA fluid, cross-linker, and photo-initiator. Then, the mixtures were polymerized at room temperature for 2 h using an ultraviolet lamp (365 nm).

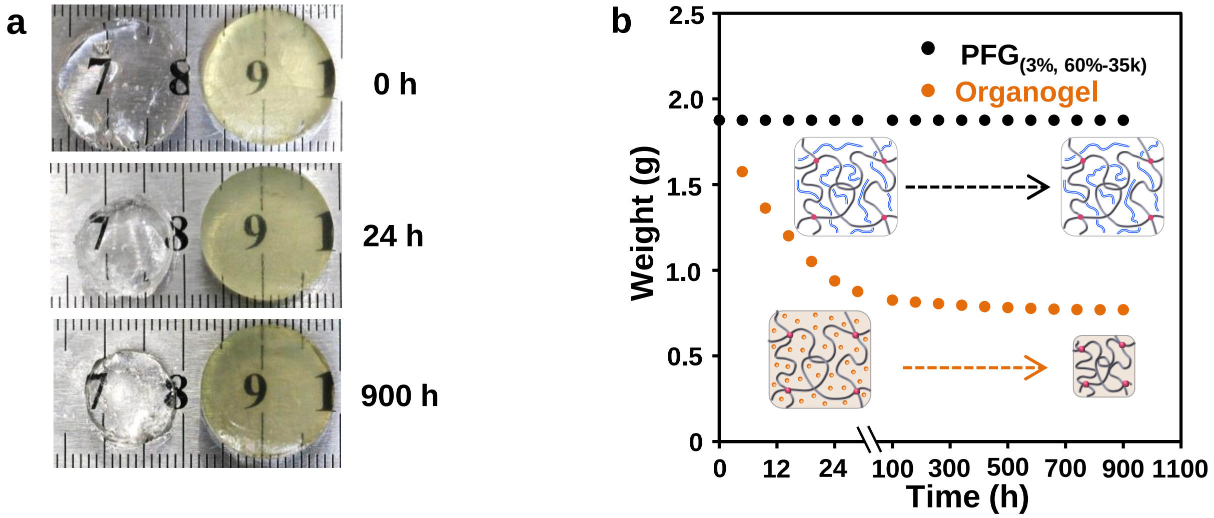

Supplementary Fig. 3 | The mass loss of PFGs. (a) The appearance of $PFG_{(3\%,\ 60\%\text{-}35k)}$ and the PBA organogel. (b) The mass lose rate of the $PFG_{(3\%,\ 60\%\text{-}35k)}$ and the PBA organogel. All samples were maintained for 900 h at room temperature.

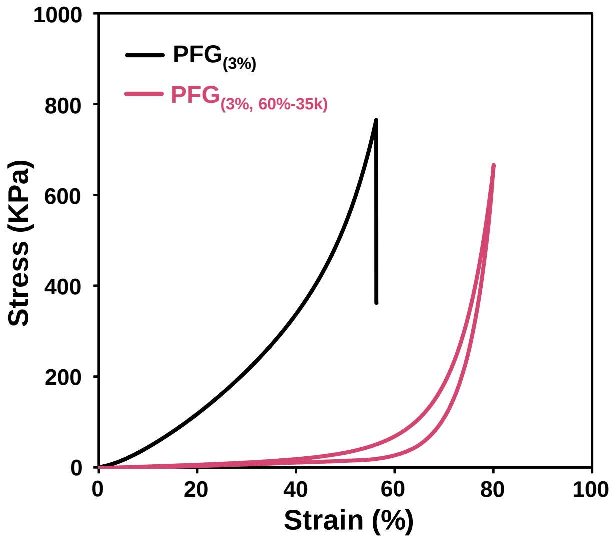

Supplementary Fig. 4 | Compressive stress-strain curves of the PFG$_{(3\%,\ 60\%\text{-}35k)}$ and the PFG$_{(3\%)}$. The PFG$_{(3\%,\ 60\%\text{-}35k)}$ exhibit better compressibility than that of the pure PBA network.

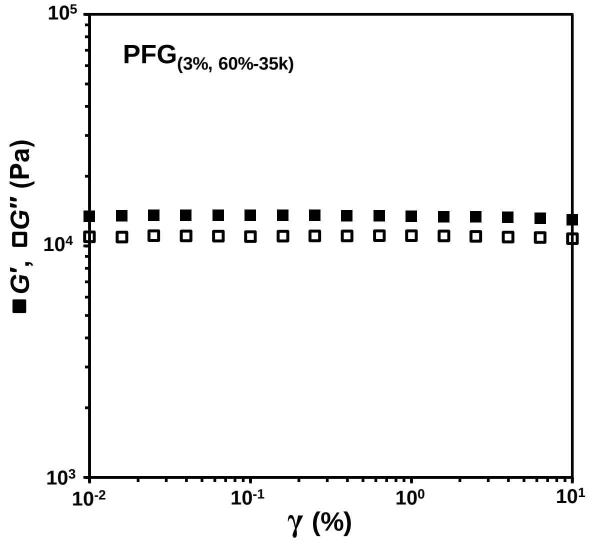

Supplementary Fig. 5 | The characterization of linear viscoelastic region. The shear strain (γ) of storage moduli (G') and loss moduli (G'') for $PFG_{(3\%,\ 60\%\text{-}35k)}$ were obtained at a constant frequency of 10 rad/s and temperature of 25 ℃. Filled squares: G', open squares: G''.

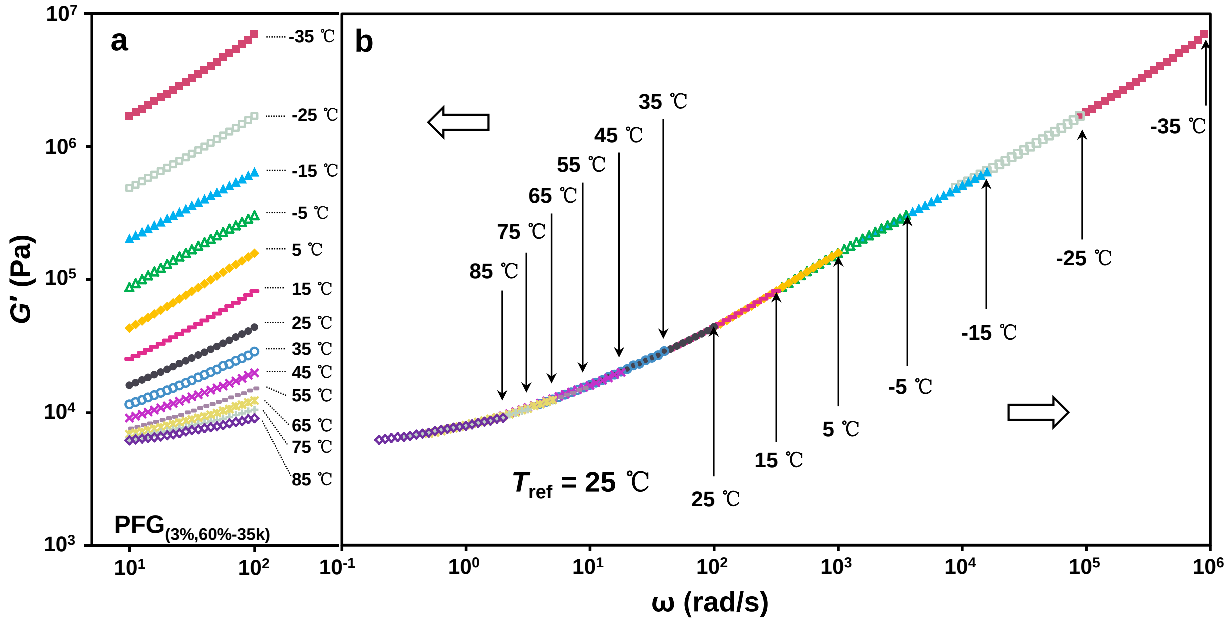

Supplementary Fig. 6 | Application of the time-temperature superposition principle. (a) *G′* data of $PFG_{(3\%,\ 60\%\text{-}35k)}$ over a $10^1 - 10^2$ Hz frequency range and a -35 − 85 °C temperature range. (b) Time-temperature master curve made from experimental data in (a) with T_{ref} = 25 °C. Filled squares: -35 °C, open squares: -25 °C, filled triangles: -15 °C, open triangles: -5 °C, filled diamonds: 5 °C, long lines: 15 °C, filled circles: 25 °C, open circles: 35 °C, forks: 45 °C, short lines: 55 °C, asterisks: 65 °C, crosses: 75 °C, open diamonds: 85 °C.

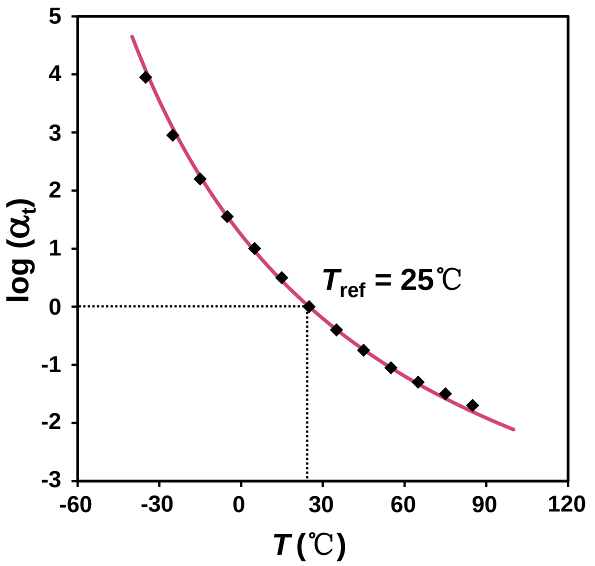

$$\log(\alpha_t) = \frac{-C_1(T-T_{ref})}{C_2+(T-T_{ref})}$$

$$T_{ref} = 25°C$$

$$T = 55°C, \log(\alpha_t) = -1.05$$

$$T = -5°C, \log(\alpha_t) = 1.55$$

$$C_1 = 6.51, C_2 = 156°C$$

$$\log(\alpha_t) = \frac{-6.51(T-T_{ref})}{156+(T-T_{ref})} \quad (1)$$

Supplementary Fig. 7 | Williams-Landel-Ferry (WLF) equation. The temperature-dependent shift factors (α_t) of the $PFG_{(3\%, 60\%\text{-}35k)}$ as a function of the temperature (black ♦) and the fitted curve of WLF equation with WLF parameters C_1 = 6.51 and C_2 = 156 ℃ for T_{ref} = 25 ℃ (red line).

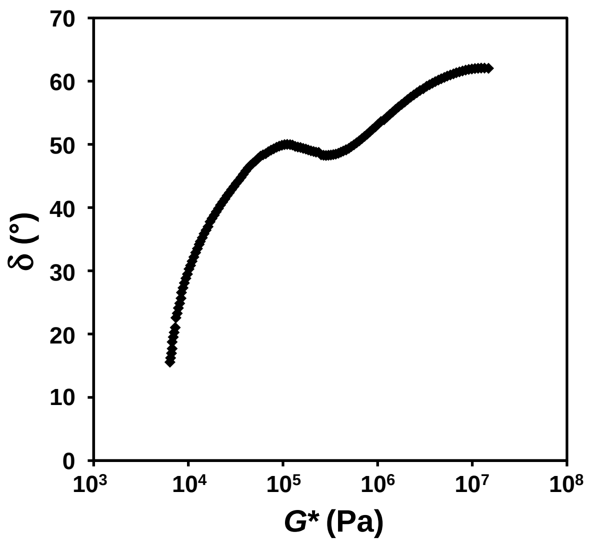

Supplementary Fig. 8 | Van Gurp-Palmen plots of PFG$_{(3\%, 60\%\text{-}35k)}$. The plots of PFG$_{(3\%, 60\%\text{-}35k)}$ are continuous, which indicates that TTS holds.

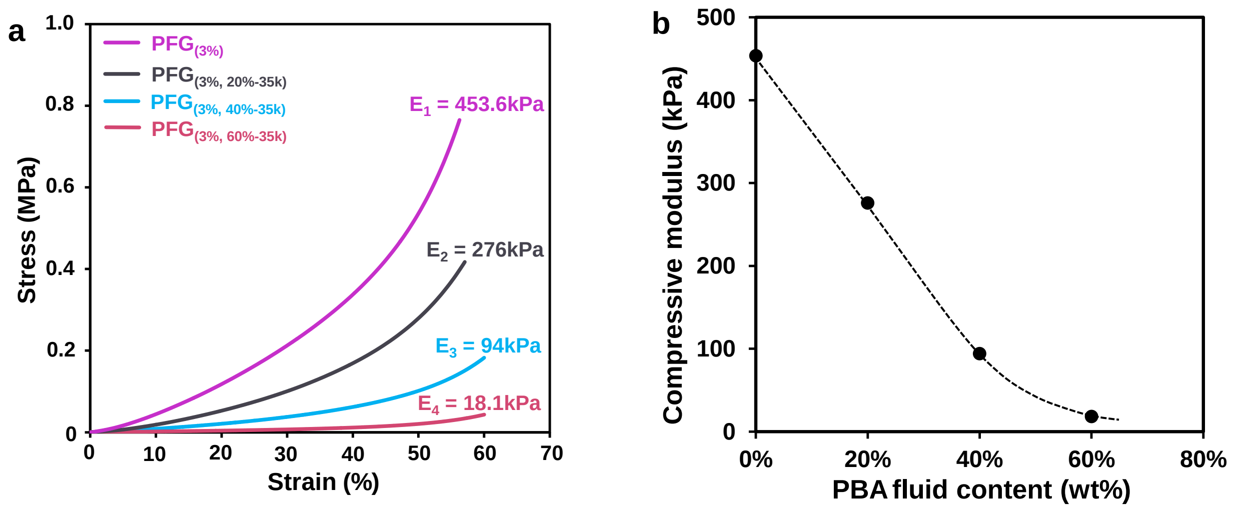

Supplementary Fig. 9 | The compressive property of PFGs. (a) Compressive stress-strain curves of the PFGs. **(b)** Compressive modulus of the PFGs with 3% cross-linker as a function of content of PBA_{270} fluid.

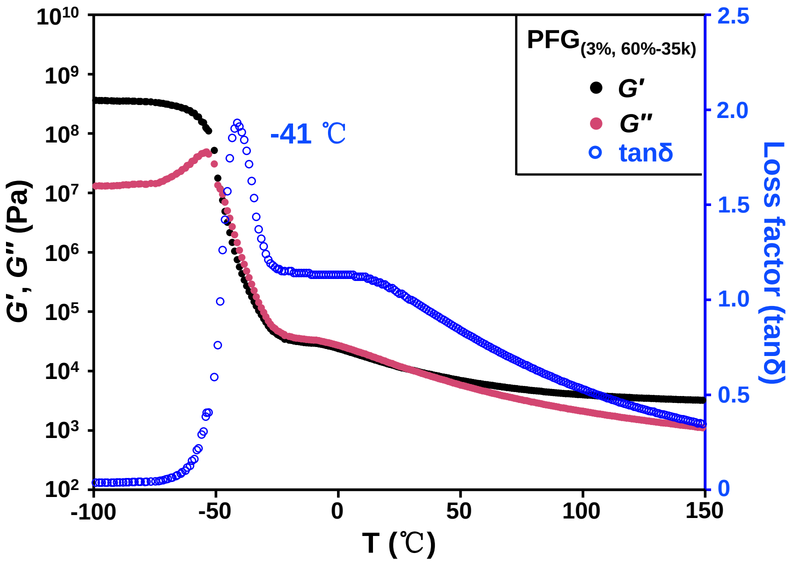

Supplementary Fig. 10 | Dynamic mechanical master curves of PFG$_{(3\%, 60\%\text{-}35k)}$. Temperature dependence of storage moduli (*G′*), loss moduli (*G″*), and loss factor (tanδ) for PFG$_{(3\%, 60\%\text{-}35k)}$ were obtained at a constant frequency of 10 rad/s with a heating rate of 2 °C/min and the shear strain of 0.5%.

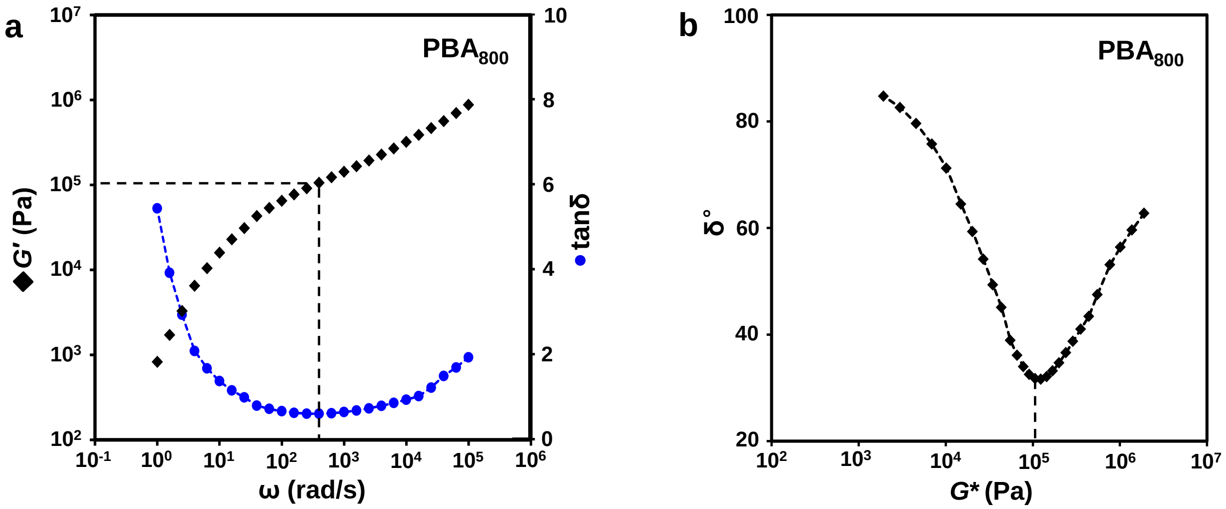

Supplementary Fig. 11 | The calculating methods of the entanglement plateau modulus of polymers. (a) minimum method and **(b)** van Gurp Palmen method, respectively. In the minimum method: the plateau modulus is taken as the storage moduli (*G′*) at the frequency corresponding to the minimum in the loss factor (tanδ). The van Gurp Palmen method assigns the plateau modulus as the storage modulus at the minimum in phase angle.

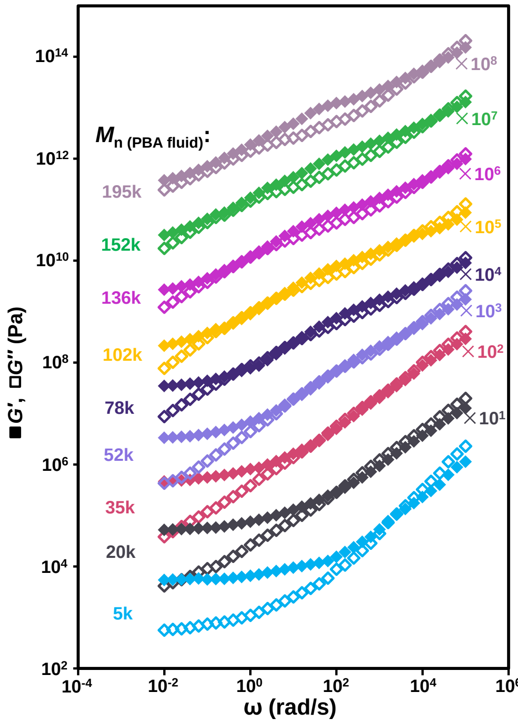

Supplementary Fig. 12 | Dynamic mechanical master curves of PFGs. Dynamic master curves of the frequency (ω) dependence of storage moduli (*G*′) and loss moduli (*G*″) were obtained at 25 °C with the shear strain of 0.5% for the PFGs containing the PBA fluid of a weight fraction of Φ = 0.6 with different molecular weights. Filled squares: *G*′, open squares: *G*″.

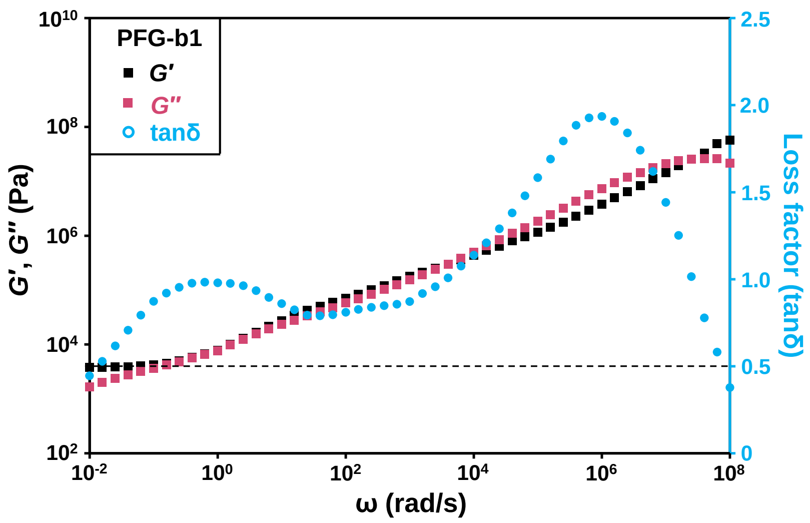

Supplementary Fig. 13 | Dynamic mechanical master curves of PFG-b1. Frequency dependence of storage moduli (G'), loss moduli (G''), and loss factor (tanδ) for PFG-b1 were obtained at 25 °C with the shear strain of 0.5%.

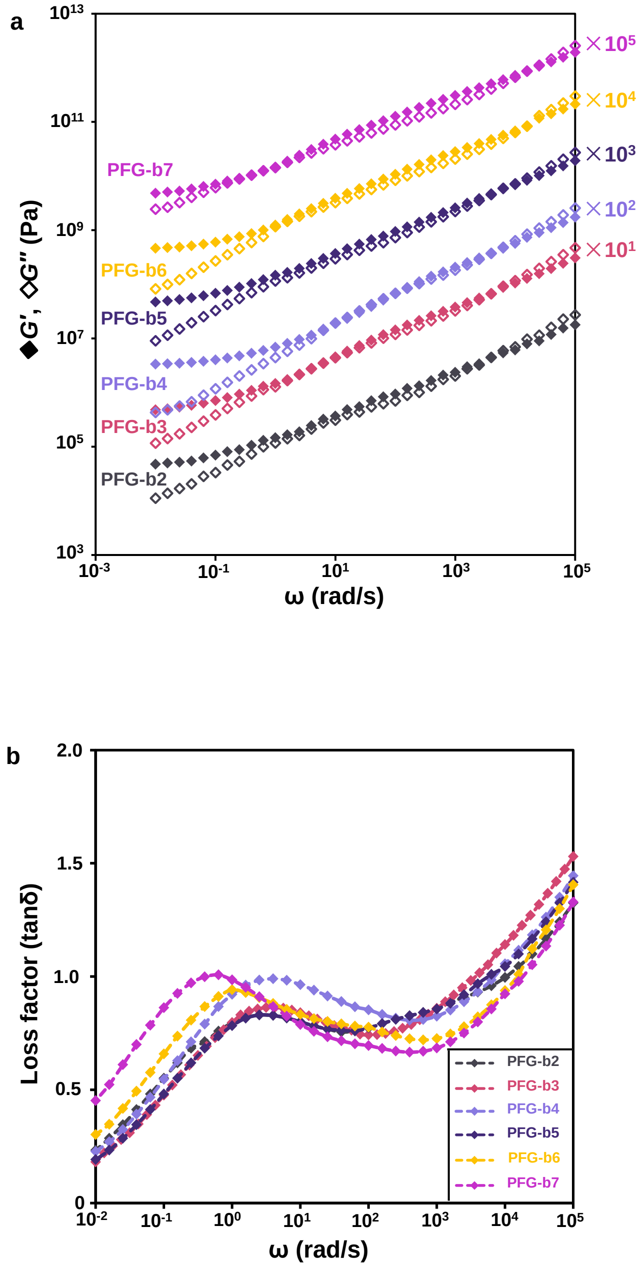

Supplementary Fig. 14 | Dynamic mechanical master curves of PFGs. Dynamic master curves of the frequency (ω) dependence of storage moduli (G'), loss moduli (G''), and loss factor (tanδ) were obtained at 25℃ with the shear strain of 0.5% for the PFGs containing the multiple PBA fluids of a weight fraction of Φ = 0.6. Filled squares: G', open squares: G''.

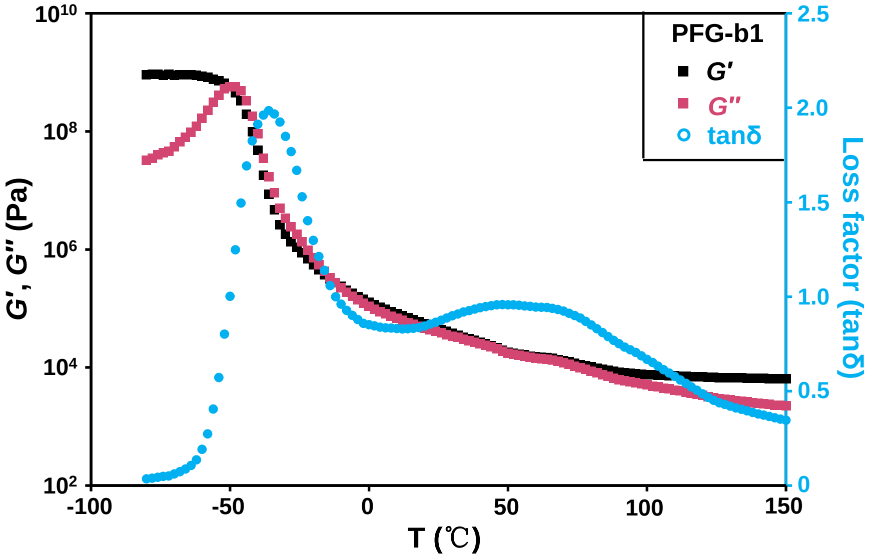

Supplementary Fig. 15 | Dynamic mechanical master curves of PFG-b1. Temperature dependence of storage moduli (*G*′), loss moduli (*G*″), and loss factor (tanδ) for the PFG-b1 were obtained at a constant frequency of 10 rad/s with a heating rate of 2 ℃/min and the shear strain of 0.5%.

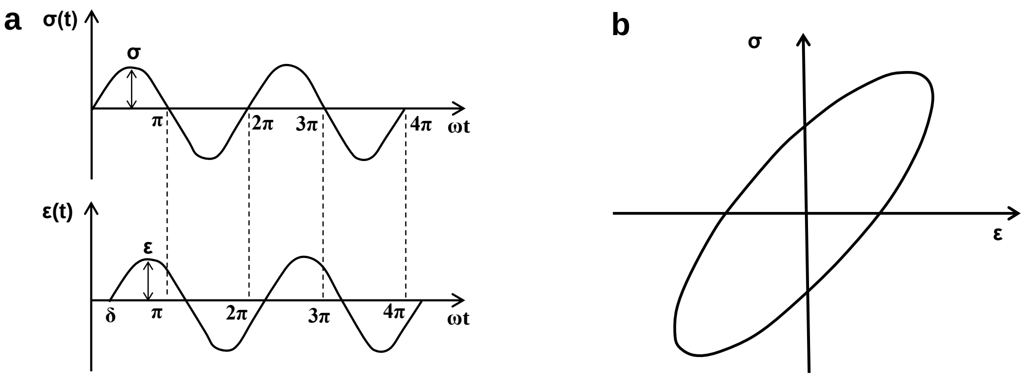

$$\sigma = \sigma_0 \mathrm{si\,n}(\omega t) \quad (2)$$

$$\varepsilon = \sigma\varepsilon_0 \mathrm{si\,n}(\omega t - \delta) \quad (3)$$

$$\Delta W = \int \sigma(t) d\varepsilon(t) = \int \sigma(t) \frac{d\varepsilon(t)}{d(t)} dt \quad (4)$$

$$\Delta W = \sigma_0 \varepsilon_0 w \int_0^{2\pi/wt} sin(wt) cos(wt - \delta) dt \quad (5)$$

$$\Delta W = \pi\sigma_0\varepsilon_0 sin\delta \quad (6)$$

Supplementary Fig. 16 | The calculating methods of the Energy dissipation (Δ*W*). When sinusoidal alternating stress (σ) was applied to the PFGs, the corresponding strain (ε) of PFGs occurred. The hysteresis loop area represents the mechanical energy that converts to heat each cycle. Energy dissipation (Δ*W*) can be calculated by the area of the hysteresis loop.

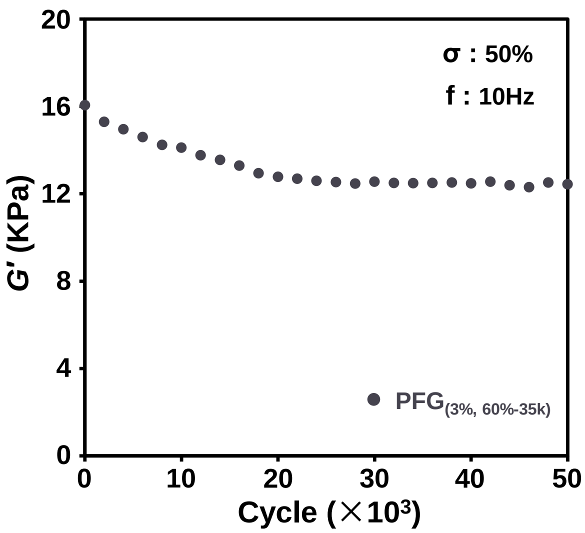

Supplementary Fig. 17 | The storage modulus (*G′*) of the $PFG_{(3\%, 60\%\text{-}35k)}$ as a function of shear cycle number. The storage modulus (*G′*) of the $PFG_{(3\%, 60\%\text{-}35k)}$ was obtained at 25℃ with the shear strain of 50% and the constant frequency of 10 rad/s.

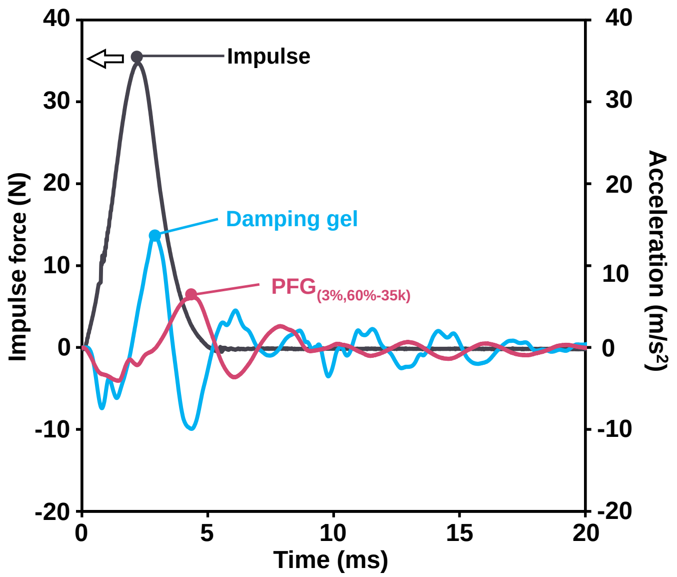

Supplementary Fig. 18 | Drop weight impact tests. The impulse force of 35 N was applied to the $PFG_{(3\%,60\%\text{-}35k)}$ and the damping gel by a drop weight. The $PFG_{(3\%,60\%\text{-}35k)}$ can absorb shock more effectively than the damping gel.

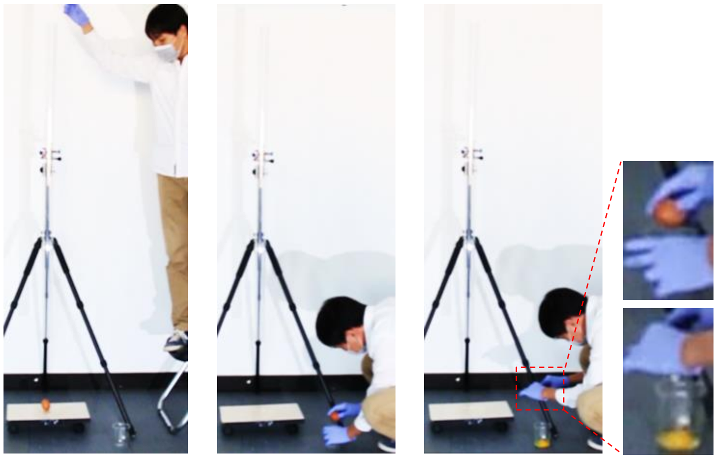

Supplementary Fig. 19 | The egg dropping experiment. The egg was dropped from 2 m height onto a 5 mm-thick $PFG_{(1\%, 60\%\text{-}35k)}$ pad and remained unbroken.

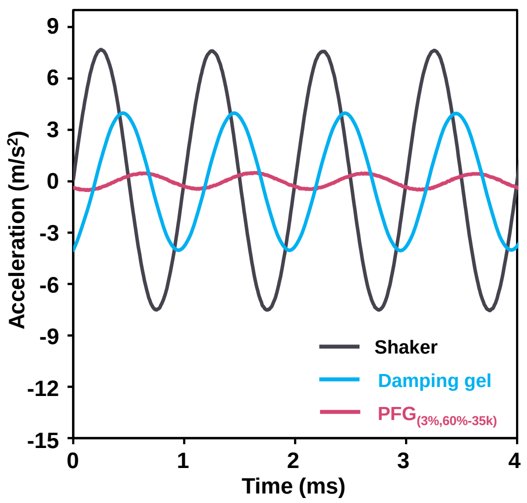

Supplementary Fig. 20 | Shaking table demonstrative experiments. The amplitude of original vibration signal with the frequency of 1,000 Hz applied to the damping gel was dissipated by 48%, whereas it applied to $PFG_{(3\%,60\%\text{-}35k)}$ was reduced by 90%.

Supplementary Tables

Supplementary Table 1. Molecular characteristics of PBA fluids.

PBA fluids	DP	M_n (kg/mol)	M_w (kg/mol)	PDI
PBA_{40}	40	5.2	6.3	1.21
PBA_{160}	162	20.3	22.1	1.09
PBA_{270}	274	35.1	37.6	1.07
PBA_{450}	454	52.2	66.8	1.25
PBA_{600}	611	78.3	93.2	1.20
PBA_{800}	798	102.2	135.9	1.33
PBA_{1000}	1014	129.8	167.4	1.29
PBA_{1200}	1184	151.6	204.7	1.35
PBA_{1500}	1524	195.1	278.9	1.43

Supplementary Table 2. Molecular characteristics of PFGs.

PFGs	$\Phi_{\mathbf{c}}$ [a] (%)	$\Phi_{\text{(PBA fluid)}}$ [b] (%)	$M_{\text{n (PBA fluid)}}$ (kg/mol)
$\text{PFG}_{(0.1\%, 60\%\text{-}5k)}$	0.1	60	5.2
$\text{PFG}_{(0.5\%)}$	0.5	0	0
$\text{PFG}_{(0.5\%, 40\%\text{-}5k)}$	0.5	40	5.2
$\text{PFG}_{(1\%, 60\%\text{-}35k)}$	1	60	35.1

[a] Weight fraction of cross-linker. [b] Weight fraction of PBA fluid.

Supplementary Table 3. Molecular characteristics of PFGs containing various PBA fluids.

PFGs	Φ_c [a] (%)	$\Phi_{(PBA\ fluid)}$ [b] (%)
		M_n (20k, 35k, 52k, 78k, 102k, 152k)
PFG-b1	3	(0, 20, 0, 0, 30, 10)
PFG -b2	3	(0, 30, 0, 30, 0, 0)
PFG -b3	3	(20, 0, 0, 40, 0, 0)
PFG -b4	3	(10, 0, 20, 30, 0, 0)
PFG -b5	3	(0, 30, 30, 0, 0, 0)
PFG -b6	3	(0, 0, 30, 0, 0, 30)
PFG -b7	3	(0, 0, 20, 0, 0, 40)

[a] Weight fraction of cross-linker. [b] Weight fraction of PBA fluid.

Supplementary Table 4. Temperature range (tanδ larger than 0.5) of the reported damping materials.

Damping materials	tanδ > 0.5 (℃ range)	tanδ > 0.5 (℃ width)	References
Chlorinated butyl rubber	-57 to 8	65	(*1*)
Styrene–butadiene rubber	-43 to -20	23	(*2*)
Gradient PEG	-30 to 18	48	(*3*)
NBR/AO-80 blend	3 to 35	32	(*4*)
Thiol−ene polymer	10 to 42	32	(*5*)
P(BA-co-MMA)	26 to 71	45	(*6*)
PU/EP IPN	38 to 100	62	(*7*)
Damping gel	-90 to -10	80	(8)

The temperature range is calculated from the frequency-dependent tanδ curves in the references.

Supplementary References

1. Zhu Y. *et al.* Study on damping mechanism based on the free volume for CIIR by PALS. *J. Phys. Chem. B* **111**, 11388−11392 (2007).
2. Wang Y. Q., Wang Y., Zhang H. F. & Zhang L. Q. A novel approach to prepare a gradient polymer with a wide damping temperature range by in-situ chemical modification of rubber during vulcanization. *Macromol. Rapid Commun.* **27**, 1162−1167 (2006).
3. D. Wang *et al.* Biomimetic gradient polymers with enhanced damping capacities. *Macromol. Rapid Commun.* **37**, 655−661 (2016).
4. Qiao B., Zhao X., Yue D., Zhang L. & Wu S. A combined experiment and molecular dynamics simulation study of hydrogen bonds and free volume in nitrile-butadiene rubber/hindered phenol damping mixtures. *J. Mater. Chem.* **22**, 12339−12348 (2012).
5. Mcnair O. D. *et al.* Impact properties of thiol-ene networks. *ACS Appl. Mater. Interfaces* **5**, 11004−11013 (2013).
6. Faghihi F., Mohammadi N. & Hazendonk P. Effect of restricted phase segregation and resultant nanostructural heterogeneity on glass Transition of nonuniform acrylic random copolymers. *Macromolecules* **44**, 2154−2160 (2011).
7. X. Lv *et al.* Damping properties and the morphology analysis of the polyurethane/epoxy continuous gradient IPN materials. *Composites: Part B* **88**, 139−149 (2016).
8. https://taica.co.jp/gel/en/product/shock_absorption/theta_sheet.html
