# Supplementary material for: Ultrahigh energy-dissipation elastomers by precisely tailoring the relaxation of confined polymer fluids
Source: Nat Commun. 2021 Jun 14;12:3610. doi: 10.1038/s41467-021-23984-2 (PMC8203694; doi:10.1038/s41467-021-23984-2)
Supplement: Supplementary file 3 — Description of Additional Supplementary Files [file 41467_2021_23984_MOESM3_ESM.docx]

Description for Additional Supplementary Files

Title: Supplementary Movie 1

Description: A supporting movie showing the tensile stress-strain test described in Fig. 2a (80×). The samples were cylinders with 6 mm diameter ×30 mm height at a displacement rate of 20 mm/min.

Title: Supplementary Movie 2, 3, 4 and 5

Description: A supporting movie showing the egg dropping experiment. An egg was dropped from 2m height onto 5 mm-thick PFG(1%, 60%-35k) pad without any cracks.

Title: Supplementary Movie 6 and 7

Description: Acoustic absorption demonstrative experiments. The damping materials with 5mm height were placed under the petri dishes containing sands and put them on an ultrasonic generator. The frequency of the ultrasonic generator was 40 kHz. The sand on the PFG is still stationary, while the sand on the natural rubber (NR) vibrates violently.
